# A mixed-methods assessment of community-engaged learning in a Master of Public Health program

**DOI:** 10.1177/20503121231176637

**Published:** 2023-05-29

**Authors:** Abhinand Thaivalappil, Rachel Coghlin, Courtney Bell, Brendan Dougherty, Stephanie Duench, Rachelle Janicki, Andrew Papadopoulos

**Affiliations:** 1 Department of Population Medicine, University of Guelph, Guelph, ON, Canada; 2 Health Promotion Team, Wellington-Dufferin-Guelph Public Health, Guelph, ON, Canada; 3 Infection Prevention and Control, Fraser Health Authority, Surrey, BC, Canada; 4 Health Protection and Investigation Division, Region of Waterloo Public Health and Emergency Services, Waterloo, ON, Canada; 5 Office of the Chief Science Officer, Public Health Agency of Canada, Guelph, ON, Canada

**Keywords:** Public health, practice-based learning, evaluation, education, community engagement

## Abstract

**Objective:**

Community-engaged learning is used in Master of Public Health programs to enhance student training, connect with communities, help solve societal issues, develop competencies, and build partnerships. However, it is unclear how much community-engaged learning components supplement existing Master of Public Health programs and prepare students in developing these competencies. Thus, the aim of this study was to apply an explanatory mixed-methods study design to evaluate a Canadian Master of Public Health program’s community-engaged learning activities and propose recommendations to strengthen public health training and course delivery.

**Methods:**

We conducted a questionnaire among Master of Public Health students (*n* = 25), focus group discussion with a subset of these students (*n* = 7), and one-on-one semi-structured telephone interviews with community partners who had previously hosted Master of Public Health students for practicum placements (*n* = 11).

**Results:**

Community-engagement enhanced learning among Master of Public Health students, with the practicum placement, and program development capstone resulting in the largest self-reported development. Students in the focus group indicated community engagement provided skill and professional development, but also identified wanting additional curriculum coverage on various statistical software and qualitative research methods. Interviews with community partners revealed benefits of practicum placements such as mutual knowledge transfer, increased organizational capacity, and strengthened academic–community partnerships. Community partners also commented on challenges with recruitment, training, and aligning student–organization goals.

**Conclusion:**

The findings from this study suggest that an update to the Master of Public Health program curriculum, its core competencies, a combination of community-engagement activities, and future evaluations will be needed to advance education delivery.

## Introduction

There is no shortage of complex public health issues and a need for professionals who can contribute to protecting and improving the health of communities.^1–3^ The coronavirus disease 2019 (COVID-19) pandemic further highlighted this critical need for public health professionals to meet the additional demands of a global crisis.^4^ In fact, students in health professions reported an increased interest in public health training and careers during the COVID-19 pandemic,^4^ emphasizing the important role that the postsecondary institutions serve in delivering public health education and preparing individuals to enter a wide range of roles centered around disease prevention and health promotion.^2^ Globally, the demand for skilled public health professionals has been expressed by the fact that many regions across the world have already developed accredited programs or are currently in the process of developing programs for public health training.^5–9^ The World Health Organization in partnership with the Association of Schools of Public Health in the European Region, has proposed a competency framework that is intended to be used for many purposes, but mainly to design curricula for professional development and degree-granting programs.^6^ The framework outlines 84 public health competencies categorized into 10 sections.^6^ Broadly, competencies are a combination of attributes, knowledge, and skills required to take on a position and outlining these competencies help a region or field guide and strengthen education.^6,10^. Competency-based programs ensure that there is an organizational structure for curriculum development, guidelines, consistency in the delivery of a high-quality training, and graduates from these programs can apply skills to deliver essential public health operations.^6,10–12^

Public health competencies vary across countries because it must serve the local public health needs of a population.^5,6,11^ Public health institutions in the United States and Canada independently outline core competencies for public health and Master of Public Health (MPH) graduates, and many of these competencies overlap.^10,13,14^ Fortunately, many core competencies (e.g., public health sciences, assessment, and analysis) can be trained through traditional course delivery methods.^2,15,16^ However, there are concerns that traditional delivery methods alone may be insufficient to develop skills to the degree required of trainees entering the public health workforce.^2,3^ Moreover, competencies highlighting the importance of skills such as strengthening partnerships, collaborating with community partners, and building community capacity to protect and promote public health may not be readily learned or experienced through conventional lectures.^10,13,17^ Based on public health’s service to community, it is generally agreed that community engagement is essential in guiding public health efforts, and is defined as the mutual exchange of resources and information between public health and community members to develop a sustained partnership.^13,17^ This sustained partnership leads to shared public health responsibility and facilitates changes in behaviors, programs, environments, and policies.^18,19^ Therefore, supplementing curricula to increase coverage of all core competencies provides the motivation for integrating community-engaged learning (CEL) within public health education.

CEL at its core involves matching students with community partners to facilitate student learning in communities where they apply knowledge and skills outside of the classroom.^13^ As a result, CEL can enhance academic outcomes, build professionals’ skills, and foster deeper academic–community partnerships that may not be possible otherwise.^13,20^ Some evidence has indicated that both community partners and students value CEL in public health.^3,13,21^ However, there may also be core competency gaps in Canada’s MPH programs,^2^ and although unclear, this limitation may be overcome by the community-engagement component of MPH programs. This lack of clarity has led to calls for investigations to evaluate public health student learning outcomes, assess community partner benefits of CEL, and explore community partner workforce needs to build and strengthen partnerships.^2,3,13^ Therefore, there is a need to rank various CEL activities and explore perspectives to cultivate a greater understanding of its ongoing effectiveness on public health training.

### Rationale

According to the World Health Organization, a strong and ready workforce that contributes to public health is one of the foundations of global sustainable development.^6^ This is translated to protecting and promoting health of communities, preventing disease, and prolonging life. A precursor to improvement of community health is public health training to meet community demands. It is believed that CEL in the form of experiential and in-service training is an important part of public health education.^13,22^ However, no study to date has extensively evaluated and published findings from multiple CEL activities in MPH programs in Canada to comment on the value that CEL has in current graduate public health training.

The objectives of this study were to conduct a mixed-methods investigation to: (a) administer a questionnaire to assess students’ self-reported change in learning outcomes after completing four CEL activities and (b) explore student and community partner perspectives of CEL through focus groups and interviews. A mixed-methods approach was used to triangulate findings and provide added depth to the quantitative component. These outputs will ultimately contribute to advancing public health by addressing the upstream prerequisites of developing a skilled public health workforce. Findings from this study will reveal strengths and gaps of CEL in public health training, identify areas for furthering public health education, and propose recommendations for strengthening academic–community partnerships.

## Methods

### Study design

This study used an explanatory mixed-methods design involving three components: (a) survey of graduate students, (b) qualitative focus group discussion with a subset of students who participated in the survey, and (c) semi-structured interviews with host agency community partners.^23^ This sequential mixed-methods approach helped to achieve the study aims through quantitative identification of the extent to which self-reported learning outcomes improved after completing CEL components, qualitative exploration of how some CEL activities may be more effective through participant discussions, and combination of the two approaches to strengthen the evaluation which provide insights into education and academic–community partnerships.

Explanatory mixed methods use a quantitative component, followed by one or more qualitative components to generate interpretations, and strengthen findings based on both components.^23^ We used a mixed-methods approach because the individual methods on their own had inherent limitations. Supplementing surveys with a focus group and interviews allows for cross-examination of results, enhance the depth and breadth of the research inquiry, prevent potentially missed results from one method only, and identify potential avenues for research and practice. In the absence of a widely accepted mixed-methods reporting checklist, we followed established reporting guidelines for qualitative research, and this is available as Supplemental Material.^24^

### Positionality and philosophical orientation

All members of the research team were affiliated with the educational institution and directly or indirectly affiliated with the MPH program at the time of data collection. All research team members were made up of men and women who were public health researchers or researchers in training. Furthermore, all were trained in quantitative and qualitative research methods. The research team had previously developed relationships with study participants, and participants were aware of the research objectives along with the public health and pedagogical interests in the study. We adopted a pragmatic worldview to approach the research question, instrument development, data collection, analysis, and reporting of this study. This worldview is compatible with mixed-methods designs and aims to use any approach, tool, or method to help answer the research objectives.^23^

### Setting

This investigation was conducted at the University of Guelph in Southwestern Ontario, Canada. The University of Guelph MPH program was formed in 2008 in response to the societal need for increased graduate-trained human resources in public health. The program was designed in compliance with the Public Health Agency of Canada’s (PHAC) Core Competencies for Public Health and guidelines for MPH programs.^10,25^ The 36 core competencies are organized under 7 categories: public health sciences; assessment and analysis; policy and program planning, implementation, and evaluation; partnerships, collaboration, and advocacy; diversity and inclusiveness; communication; and leadership (PHAC, 2008). The competencies form a set of knowledge, skills, and attitudes to be practiced by professionals within the public health sector.

The MPH program at the University of Guelph uses multiple CEL opportunities: (a) a 12-to 16-week practicum placement where students work with and support a public health organization by focusing on a major project; (b) an application of public health practice tools in-class for case studies or assignments; (c) a public health capstone course which involves the development of public health materials, knowledge translation, and presenting practicum placement outcomes to peers and public health professionals; and, (d) an applied public health research component which is a supervised research project where the student investigates a public health issue, performs analysis, interprets health information, and prepares a final report.

### Participants

To be eligible, all participants were older than 18 years of age and had to be registered MPH students or community partners who were currently hosting or had previously hosted MPH students for a practicum placement. All eligible students were active full-time or part-time graduate students of the MPH program to meet our inclusion criteria. Alumni and future students were not eligible to participate. Community partners were identified through a living database that was managed by the MPH Program Coordinator. To be eligible to participate, community partners had to be employed at the organization at which they hosted MPH students during the data collection period. Community partners were also required to oversee some or all aspects of MPH students’ practicum placement to be eligible for inclusion.

### Recruitment and data collection

Data collection was conducted between December 2016 and January 2017. This period was selected because it marked the end of the fall term and the beginning of the winter term, and therefore, concluding practicum placements or beginning new placements. The surveys, focus group discussion, and in-depth interviews were conducted in English.

A purposive sampling approach was employed based on the inclusion and exclusion criteria. Recruitment emails were sent to all active MPH students who had recently participated in ⩾1 of the following: public health practicum, applied public health research course, public health administration course, or in-class case studies. The email included a letter of information outlining the scope of the project, participants’ rights, confidentiality, and details on consent. Embedded within the recruitment email were links to four online surveys hosted by Qualtrics (Qualtrics International Inc., Provo, UT, USA). Individuals completed a series of questions regarding their practicum experience (e.g., During your practicum placement, what did your primary role involve?), proficiency in core competencies (e.g., Rank your competence and ability to identify and collaborate with partners in addressing public health issues), and other components of CEL (e.g., Did this style of learning improve retention of in-class content?). The questionnaire and other data collection instruments used in this study were adapted from previously developed unpublished internal question guides, pretested on approximately 10% of the targeted study population, and later refined through discussion. The estimated time for completion of each survey was 20–30 min, all responses were anonymous, and a copy of the questionnaire is provided as Supplemental Material.

Respondents from the survey were invited to participate in focus group discussions on campus. Individuals who indicated interest were provided information on participating and consent. Upon arrival to the focus group, participants were made to read details on consent and asked to sign a consent form. This session lasted for 1 h and was moderated by one member of the research team, with three others co-moderating and writing notes during the session (RC, CB, BD, SD). During the session, students were asked to comment on their involvement and experience in any four of the CEL activities delivered by the MPH program. Due to confidentiality purposes and to protect participants, we opted to write notes throughout the discussion on key topics rather than record the audio of the session. Therefore, no transcribing was conducted and data collection was informed by a less intrusive note-taking approach by focus group co-moderators. Only one focus group discussion was carried out due to low student interest in this component. A similar study exploring MPH students’ perspectives about education delivery revealed that depth and data saturation was met with just six participants.^5^ According to previous research, there is no consensus on the number of participants needed for qualitative research studies and it is suggested that thematic or data saturation should be emphasized to ensure that all key points of discourse are covered.^26,27^ Therefore, we aimed for data saturation across the focus group and one-on-one interviews. Focus group moderators and interviewers were previously trained in qualitative data collection techniques. A copy of the focus group question guide is available as Supplemental Material.

After completion of the focus group, an email was sent to community partners who had hosted University of Guelph MPH students on multiple occasions. Agencies who agreed to a one-on-one telephone interview were contacted by the research team via email to confirm a date and time and were asked to provide written consent prior to the start of the interview. Interviewees were reminded that only notes would be written by the interviewer, calls would not be recorded and therefore no transcripts would be made available, and their responses would remain anonymous. All interviewees answered a series of questions regarding their experience in hosting graduate students for practicum placements and their perspectives on CEL. Both data and thematic saturation of qualitative interviews and the focus group discussion were reached early on, as there were no contentious topics or substantially different experiences. Telephone interviews ranged between 10 and 15 min, and a copy of the interview guide is accessible as Supplemental Material.

### Statistical analysis

Survey responses were exported from the online survey platform and imported to Microsoft Excel (Microsoft, Redmond, WA, USA). Each respondent was asked to answer the same question both before and after their CEL experience regarding their subjective level of skill in the selected core public health competencies (i.e., public health sciences; assessment and analysis; policy and program planning, implementation, and evaluation; partnerships, collaboration, and advocacy; diversity and inclusiveness; communication; and leadership). Responses were in Likert scale (i.e., poor, average, good, excellent, and not applicable) and assigned a value between 1 and 4.^28^ Any “not applicable” responses were excluded from the analysis. Descriptive statistics were evaluated to summarize data. Next, Wilcoxon matched-pairs signed-rank tests were performed, and the difference in mean ranks were estimated to assess potential changes in respondents’ self-reported core competencies before and after completing a CEL activity. This test statistic was selected because we were analyzing nonparametric paired data.^29^ We calculated a minimum required sample size of 21 which was based on previously established sample size calculations and the following parameters: α = 0.05, power of 90%, detecting *p*’ of 0.85, and no ties.^30^ Total survey respondents recruited for this study was 25, which exceeded the required minimum. All statistical analyses were performed using STATA/SE 14 (StataCorp, College Station, TX, USA).

Qualitative data were analyzed by four members of the research team (RC, CB, BD, SD, RJ). Some elements of reflexive thematic analysis were borrowed which involved the following steps: data familiarization process, a semantic approach to coding, and peer debriefing of salient topics that arose during discussions from the focus group and community partner interviews.^31^ We intended to descriptively summarize qualitative findings and generate themes that encompass prominent and recurring topics across students and community partners. Trustworthiness of focus group qualitative findings was enhanced by including some members of the research team who were MPH students at the time of data analysis. Member checking was not employed for the analysis of community partner interviews. No statistical software was used in the analysis of qualitative data.

### Ethics

Written informed consent was collected from all participants in the respective surveys, focus group discussion, and semi-structured interviews. Furthermore, survey responses were anonymized and could not be traced back to the original respondent to protect the participant and reduce the likelihood of social desirability bias. Although the quantitative component used an anonymous online survey format, we acknowledge that participants from the qualitative components may have provided more conservative or favorable responses due to preexisting relationships between researchers and study participants. To promote candid response, all individuals were reminded verbally and in writing that participation in this study would not affect their relationship with the academic institution, MPH Program Coordinator, and that their responses would not be traced back to them. This study was reviewed and approved by the Research Ethics Board at the University of Guelph (REB#16-JL-037).

## Results

### Self-reported measures and student perspectives

Overall, 25 out of the 50 MPH students contacted responded to the survey, which resulted in a 50% response rate and meeting the minimum required sample size for this study. Although demographics for the student survey were not collected, the typical MPH student cohort for this institution in recent years has predominantly identified as women, White, and under 30 years old (>50%). Generally, all four CEL activities improved graduate students’ self-reported self-efficacy, problem-solving, critical thinking, leadership skills, interpersonal skills, verbal communication, and written communication. Horizontal bar charts depicting self-reported skill development after the public health practicum (Supplemental Figure A1), in-class case studies (Supplemental Figure A2), program development capstone assignment (Supplemental Figure A3), and applied public health research component (Supplemental Figure A4) can be accessed in the Supplemental Material.

Tables 1 and 2 show results for the most and least improved core competencies after completion of the four CEL activities (i.e., public health practicum, program development capstone assignment, in-class case studies, and applied public health research). The rank column depicts the three largest improvements in core competencies (Table 1) and the three smallest improvements in core competencies (Table 2). The mean signed-rank differences take into account the magnitude of the observed differences before and after each CEL component, ranking them based on smallest to largest, and assigning a sign to indicate whether the CEL component had a positive or negative impact on acquiring core competency skills. The final column shows core competencies, which are the key required skills outlined by the government that MPH trainees will use in the workforce. As indicated earlier, there are a total of seven competency categories: public health sciences; assessment and analysis; policy and program planning, implementation, and evaluation; partnerships, collaboration, and advocacy; diversity and inclusiveness; communication; and leadership.

**Table 1.** Self-reported core competencies to have improved the most (*n* = 3) after completion of CEL opportunities by MPH students.

Learning component	Rank	Mean signed-rank difference	Core competency^a^
Public health practicum	1	15.44	Public health sciences (1.3)
	2	14.07	Public health sciences (1.4)
	3	13.10	Assessment and analysis (2.6)
Program development capstone assignment	1	9.49	Policy and program planning, implementation, and evaluation (3.3, 3.4)
	2	9	Assessment and analysis (2.2)
	3	8.23	Public health sciences (1.5)
In-class case studies	1	7.67	Policy and program planning, implementation, and evaluation (3.6)
	2^b^	6.56	Public health sciences (1.1); partnerships, collaboration, and advocacy (4.4)
	3	6.33	Public health sciences (1.2)
Applied public health research	1	3.88	Assessment and analysis (2.6)
	2	3.71	Assessment and analysis (2.4)
	3	3.50	Public health sciences (1.4)

a Numbers under this column denote specific public health core competencies outlined under Core Competencies for Public Health in Canada.

b This level contains two competency categories because both were estimated to have the same mean signed-rank difference value.

**Table 2.** Self-reported core competencies to have improved the least (*n* = 3) after completion of CEL opportunities by MPH students.

Learning component	Rank	Mean signed-rank difference	Core competency^a^
Public health practicum	1	3.64	Policy and program planning, implementation, and evaluation (3.8)
	2	3.83	Leadership (7.5)
	3	5.77	Policy and program planning, implementation, and evaluation (3.2)
Program development capstone assignment	1	1.9	Policy and program planning, implementation, and evaluation (3.8)
	2	3.67	Leadership (7.3)
	3	3.85	Diversity and inclusiveness (5.3)
In-class case studies	1	2.22	Communication (6.1)
	2	2.25	Leadership (7.5)
	3	2.67	Communication (6.2)
Applied public health research	1	−0.23	Policy and program planning, implementation, and evaluation (3.8)
	2	−0.17	Diversity and inclusiveness (5.2)
	3^b^	0	Communication (6.1), Leadership (7.5)

a Numbers under this column denote specific public health core competencies outlined under Core Competencies for Public Health in Canada.

b This level contains two competency categories because both were estimated to have the same mean signed-rank difference value.

Overall, the public health practicum and capstone assignment experienced the largest self-reported development in their respective competency categories. The three largest increases are shown in Table 1. Of the seven core competency categories, the most improved competencies across CEL activities were only seen in four categories: (a) public health sciences; (b) assessment and analysis; (c) policy and program planning, implementation, and evaluation; and, (d) partnerships, collaboration, and advocacy (Table 1).

In contrast, in-class case studies and applied public health research had the lowest improvement scores (Tables 2). Of the seven core competency categories, the least improved competencies across CEL activities were in four categories: (a) policy and program planning, implementation, and evaluation; (b) leadership; (c) communication; and, (d) diversity and inclusiveness (Table 2).

In total, seven students from the survey agreed to participate in the focus group discussion. Participants reported working for academic settings, nongovernmental public health organizations, local public health units, and provincial and federal government. Their roles were focused on areas such as health communication, epidemiology, knowledge translation, and qualitative research methods. We mapped salient points from the focus group discussion to themes shown in Table 3.

**Table 3.** Themes generated from the focus group discussion with MPH students (*n* = 7).

Theme	Key points
Practicum as a positive experience that advances learning, with varying student–supervisor relationships	• It was an enjoyable experience to work with community partners, but it took time to develop strong, open communication with supervisor(s). • Some stated their supervisor did not have enough time for open communication, while others had maintained a working relationship with the organization and the supervisor even after the practicum. There were instances of incongruent student–supervisor expectations on responsibilities. • Work during the placement was taken more seriously because it had the potential to impact public health (versus grades only). • Learning was accelerated because it was a short timeframe with high expectations and people depended on them. Students retained what they learned on the job due to the practical nature of applying skills and knowledge.
An effective tool for academic and professional development	• Practicums provided an opportunity to apply course content to community settings, gain a stronger understanding of the research process, guide future academic interests, and learn public health content not covered in classroom sessions. • Practicums facilitated an enhanced curriculum vitae, and networking opportunities through conferences and professional events. • Students learned about their own public health interests, conflict resolution, improved self-confidence, and building professional relationships with colleagues.
Self-realization of the preparedness and value that the trainees bring to the community	• Students realized they brought specific skills to the workplace, such as technology, software prowess, and ability to mentor others. • Information learned through prerequisite courses sometimes surpassed knowledge expectations of community partners.
Limitations of community engagement and gaps in the curriculum	• It was impossible to obtain all core competency skills during the timeframe of the practicum placement. Students wished to advance their core competencies in leadership, program evaluation, public health sciences, and analytical skills. • Skill and knowledge gaps were reported in qualitative research methods, statistical software (e.g., SPSS, SAS, Excel), multivariable analyses, and dissemination of public health materials. • A need to reduce the emphasis of some components (e.g., environmental health) to allow exploration of other public health interests and courses.

### Community partners’ experiences

Of the 14 community partners contacted for interviews, 11 agreed to participate. This component had a response rate of 79%. Community partners were employed in government, university settings, or nongovernmental public health organizations across Canada (Table 4). All interviewees reported having hosted one or two MPH students during the last practicum period. Community partners had indicated that their practicum students typically held positions in epidemiology, health communication, health promotion, knowledge translation, and program evaluation. The interview guide prompted responses from these community partners, with salient and recurring topics being mapped onto themes in Table 5.

**Table 4.** Demographic characteristics of community partners interviewed (*n* = 11).

Demographic characteristics	*N*	%
Gender		
Man	1	9.1
Woman	10	90.9
Represented agency		
Public Health Agency of Canada	4	36.4
Wellington-Dufferin-Guelph Public Health	2	18.2
Halton Public Health	2	18.2
Cancer Care Ontario	1	9.1
Ontario Ministry of Agriculture, Food and Rural Affairs	1	9.1
University of Guelph Wellness Centre	1	9.1

**Table 5.** Themes generated from semi-structured telephone interviews with community partners (*n* = 11).

Theme	Key points
Empowers students and presents mutual benefits to organizations	• Practicums provided students with increased confidence, knowledge, productivity, and ability to collaborate and contribute to a team environment. • Students developed in many areas such as presentation skills, data analysis, ability to ask the right questions, report writing, verbal communication, and project management. • Organizations in turn experienced increased work capacity to complete tasks that normally did not have the time to be completed and access to additional resources.
Two-way knowledge transfer and enhances academic–community partnership	• Supervisors acknowledged that there was mutual knowledge transfer. Specifically, community partners learned new and different approaches from students (e.g., technology use, statistical analysis). • There was an exchange where the supervisor learned how to support and mentor students, and in return, students gained skills from the supervisor. • Agencies who recently began hosting students stated that they felt a strengthened university–agency relationship.
Showcases the importance of practicum placements in addition to in-class teaching methods	• Placements provided hands-on learning experience, prepared students to enter the workforce, provided an opportunity to further develop and refine skills, increased knowledge retention of previously taught concepts, and taught how to adapt to changing priorities in a less-structured environment. • Practicums also offered intangible benefits such as networking, ability to consult with people from different disciplines, workplace etiquette, and learning to work well with others.
Presents logistical challenges associated with the organization and seeking high-quality candidates	• A logistical disadvantage was recruitment and training were time consuming aspects of onboarding a practicum student—this was resource intensive for only having a student for a short period of time. • It was challenging to ensure the needs of the agency aligned with the needs of course components and/or students sometimes. • The following elements were considered important in practicum students: open communication, ongoing feedback, shows interest in hosts’ project, mutual respect, general good employee traits (e.g., punctual, personable, proactive, strong work ethic), and acknowledges mutual needs and expectations.

Across the student focus group discussion and community partner interviews, many aspects of the practicum placement blended and both parties acknowledged the benefits of various elements such as real-world experience, professional development, public health skill development, and mutual knowledge transfer. They also realized the limitations of a 4-month practicum in accomplishing larger project-oriented and skill-oriented goals.

## Discussion

This mixed-methods study provided evidence from three methodological approaches and two distinct groups that CEL enhances learning outcomes, furthers the development of several public health core competencies, increases organizational capacity, and promotes mutual knowledge transfer. Regarding general skills, students reported that all four CEL activities improved self-efficacy, problem-solving, written communication, verbal communication, interpersonal skills, critical thinking, and leadership. Students and community partners from the qualitative component expressed several benefits from CEL, but also specified needs such as financial compensation, longer practicum placements, and refreshing course curriculum. Previously published literature has typically focused on students, community partners, or one CEL component.^4,13,16^ To our knowledge, this is the first study of its kind to explore the advantages of various CEL opportunities in combination from student and community partner perspectives. Employing mixed methods allowed us to corroborate findings and generate greater depth of findings that likely would have not been possible with one method alone.

Regarding core competencies, students in our study reported the largest improvements in areas of policy and programs, public health sciences, and assessment and analysis. On the other hand, they also reported some learning gaps in categories related to communication, leadership, and diversity and inclusiveness. This may be partially attributed to former categories being activity-based compared to leadership and communication which are general professional skills. These latter skills may have been previously developed through other extracurricular activities or prior to entering the MPH program, leading to smaller perceived learning gains in these categories. Other studies highlight the value of communication and leadership and note that these are extensively used during the MPH program and in the workplace,^16,32,33^ indicating a need to identify strategies to improve the magnitude of learning in these areas.

The focus group discussion identified barriers, needs, and curriculum preferences (e.g., qualitative methods, statistical software, changes in mandatory course requirements) in addition to the gaps in leadership, communication, and diversity and inclusiveness. Two studies assessing Canadian MPH programs arrived at similar conclusions where authors found that program curricula emphasized public health sciences compared to other competency categories and new paradigms.^2,15^ In 2021, the Chief Public Health Officer’s Report on the State of Public Health included an acknowledgment that competencies could be added or enhanced to include categories such as community engagement, and social and racial equity among others.^34^ It is clear, at least in Canada, that MPH programs and core competencies must be reassessed and perhaps refreshed to meet the needs of students and current public health workforce demands.^2,15^

Regarding CEL activities, the largest self-reported core competency improvements among students in our study were experienced through the public health practicum (versus capstone assignment, in-class studies, and applied public health research). This was highlighted in the fact that most conversations from the focus group and interviews revolved around the practicum placement. Typically, a public health practicum in Canada lasts 12–16 weeks and requires students to work full time with a community partner. This immersive experiential learning component may facilitate increased frequency and magnitude of core competency development compared to other CEL activities. However, the duration and immersion of CEL activities vary, making them challenging to compare. Based on our study findings, it appears that all four components studied demonstrate benefits in slightly different competencies. Previous research assessing MPH program’s learning outcomes reported increases across many topics and competencies.^16,35^ However, these studies also vary in the outcomes and competencies studied, making it challenging to corroborate findings. Regardless, it is unlikely that any single practice-based or in-class course component effectively results in knowledge acquisition and confidence in all public health core competencies. Rather, each CEL can focus on only a few competencies, and thus, participation in multiple valuable CEL experiences can allow students to gain skills across different competencies to ensure strong education, student satisfaction, academic–community linkages, and professional development.^3,21^ Further research is needed to identify how CEL activities address core competencies differently and which combination(s) of CEL opportunities result in the most comprehensive coverage of skills and public health core competencies.

In the 1996 seminal essay, Ernest Boyer stated that scholarship of engagement involved connecting academia with society, continuously and creatively collaborating with each other to answer important social, civic, economic, and moral problems.^36^ It is this engagement that contributes to community-engaged scholarship and mutual academic–community benefit.^13,36^ Similar to our findings, other studies show that CEL improves skill development among students, networking, organizational capacity, and deepens academic–community relationships.^13,37^ The public health practicum’s value is through experiential learning that results in the commitment to community, and others advocate for the benefits of integrating community engagement within public health training.^3,13,15,21^ Although CEL was perceived as successful by all parties in our study, some community partners identified needs such as extending the period of placements to ensure project completion and challenges in aligning student needs with the organization. Academia can demonstrate its commitment to the community and strengthen existing relationships by integrating periodic student and community partner assessments throughout practicum placements. These evaluations can identify ongoing student–community challenges to propose proactive solutions or program-wide changes to meet community needs and better prepare the next generation of public health professionals.

### Implications for the broader geographic context

Public health competencies vary across countries because skills and attributes need to be context specific and cater to the demands of the local populations.^38,39^ For instance, competencies from low- and middle-income countries may not apply to high-income countries.^9^ Globally, several regions have adopted their own public health competencies or are in the process of drafting them to support MPH and other public health training.^7,9,11,38–41^ Generally, there are many shared competencies across countries such as policy planning and development, leadership, collaboration, communication, advocacy, and systems thinking.^6,10,40^ These make up the core public health skills and attributes necessary to create a unified and strong public health workforce. The present study only evaluated change in competencies after CEL activities from a program in Canada, but some of these findings may also apply to a broader context. For instance, a multicountry study assessing MPH programs in China, Mexico, the Netherlands, South Africa, Sudan, and Vietnam revealed that MPH graduates who indicated that their graduate program enabled them to substantially apply various core competencies ranged between 33% and 48% with policy development competencies being rated the lowest.^35^ Furthermore, a qualitative study from the United Kingdom exploring students’ experiences found that MPH students wanted more options to increase their employability.^5^ This suggests that current program curricula may not be achieving optimally intended competency-based training for public health graduates. Combined with the findings from our study which revealed that each of the CEL activities provided discrete benefits of: (a) general work-related skills such as critical thinking, problem-solving, and self-efficacy and (b) core competencies such as policy, leadership, and public health sciences, it is likely that multiple CEL activities must be integrated into MPH programs to supplement traditional course delivery.

### Recommendations for public health training

CEL helps to further student learning, address community concerns, may improve departmental scientific rigor, and enhance applied research in public health.^13^ We found that CEL activities improved self-reported learning outcomes, and each CEL activity favored certain core competencies. Our recommendations include amendments to curriculum, CEL, and evaluations. First, public health curricula can consider the following to advance graduate training: implementing a mandatory community-engagement component, encouraging faculty to adopt CEL into their teaching strategy, refreshing MPH programs to implement current workforce needs (e.g., qualitative methods), and increasing coverage of competency gaps (i.e., leadership, communication, diversity, and inclusiveness) by integrating them into existing course offerings. Second, our study provides further rationale for increasing the emphasis on community engagement in MPH programs,^15,42^ overcoming barriers faced by community partners who currently participate or intend to participate in CEL,^13^ coordinating multiple extended CEL opportunities to ensure development of various core competencies, and periodically assessing CEL activities to identify areas for improvement to achieve a well-balanced graduate training that covers all core competencies. Lastly, investing in infrastructure to conduct ongoing evaluations of public health program components, CEL activities, and entire MPH programs can promote knowledge transfer and strengthen education delivery especially if they are publicly disseminated.^3,13^ Using a combination of these may overcome existing teaching and learning core competency gaps, strengthen academic–community partnerships, and identify current student- and workforce-specific needs to further public health training and quality of education delivery.

### Limitations

First, the study draws on data from a small sample size using non-probability sampling, which has the potential to invite biases. However, the aim of this study was to evaluate learning outcomes and explore community-engagement approaches to education rather than establish generalizable parameters. Furthermore, we reached minimum sample size requirements in the survey component, and thematic saturation in both the focus group and one-on-one interviews. The mixed-methods assessment allowed to overcome some of the individual component limitations, facilitated cross-comparison of these results, and strengthened the overall findings. Secondly, we used anonymous responses in the survey methods, but the qualitative components required direct interaction between the research team and study participants. However, this also enhanced our ability to collect extensive data and facilitate richer discourse in the focus group and interviews. Thirdly, we did not record any audio from the focus group and telephone interviews due to confidentiality and to promote open responses, but as a result, there were no illustrative quotes to support qualitative findings. Future investigations should attempt to invite participant voices while still protecting anonymity to build on this study. Lastly, the data were collected before the COVID-19 pandemic and there are some concerns about external validity and whether the findings are generalizable beyond the context and duration of the study. However, many, if not all, of the study findings may still apply today because there have been no changes made to the Canadian public health core competencies in nearly 15 years. Regardless, an update to this assessment may provide insights into how CEL has been impacted during the COVID-19 pandemic, and how training can respond to public health crises.

## Conclusion

This mixed-methods study assessed MPH students’ self-reported student learning outcomes, experiences with multiple community-engagement components, and triangulated findings through community partner interviews. Students reported large improvements in some competency categories such as public health sciences, and gaps in others such as leadership. Students and community partners experienced several benefits from community-engagement learning (e.g., mutual knowledge transfer, networking, increased organizational capacity); but also identified needs (e.g., financial compensation for students, longer placements, updating course curriculum). This study demonstrates the effectiveness of using a combination of community-engagement learning activities to increase coverage of public health competencies.

Going forward, MPH graduate curricula and existing public health core competencies must be revisited and modified to enhance learning outcomes and create a prepared workforce. Workforce demands, community needs, and student expectations must also be considered when making changes to the curricula. We recommend future research explore solutions to the student and community barriers identified in this study and publish evaluations of public health programs in other institutions and regions.

## Supplemental Material

sj-doc-1-smo-10.1177_20503121231176637 – Supplemental material for A mixed-methods assessment of community-engaged learning in a Master of Public Health program Click here for additional data file. Supplemental material, sj-doc-1-smo-10.1177_20503121231176637 for A mixed-methods assessment of community-engaged learning in a Master of Public Health program by Abhinand Thaivalappil, Rachel Coghlin, Courtney Bell, Brendan Dougherty, Stephanie Duench, Rachelle Janicki and Andrew Papadopoulos in SAGE Open Medicine

sj-docx-2-smo-10.1177_20503121231176637 – Supplemental material for A mixed-methods assessment of community-engaged learning in a Master of Public Health program Click here for additional data file. Supplemental material, sj-docx-2-smo-10.1177_20503121231176637 for A mixed-methods assessment of community-engaged learning in a Master of Public Health program by Abhinand Thaivalappil, Rachel Coghlin, Courtney Bell, Brendan Dougherty, Stephanie Duench, Rachelle Janicki and Andrew Papadopoulos in SAGE Open Medicine
