# Supplementary material for: A mixed-methods assessment of community-engaged learning in a Master of Public Health program
Source: SAGE Open Med. 2023 May 29;11:20503121231176637. doi: 10.1177/20503121231176637 (PMC10233587; doi:10.1177/20503121231176637)
Supplement: sj-doc-1-smo-10.1177_20503121231176637 – Supplemental material for A mixed-methods assessment of community-engaged learning in a Master of Public Health program [file sj-doc-1-smo-10.1177_20503121231176637.doc]

**Consolidated criteria for reporting qualitative studies (COREQ): 32-item checklist**

Tong A, Sainsbury P, Craig J. Consolidated criteria for reporting qualitative research (COREQ): A 32-item checklist for interviews and focus groups. *International Journal for Quality in Health Care*. 2007. Volume 19, Number 6: pp. 349 – 357

| **Item** | **Guide questions/description** | **Reported location** |
| --- | --- | --- |
| **Domain 1: Research team and reﬂexivity** | | |
| *Personal Characteristics* | | |
| 1. Interviewer/facilitator | Which author(s) conducted the interview or focus group? | Page 5 – recruitment and data collection |
| 2. Credentials | What were the researcher’s credentials? e.g., PhD, MD | Page 4 – positionality and philosophical orientation |
| 3. Occupation | What was their occupation at the time of the study? | Page 4 – positionality and philosophical orientation |
| 4. Gender | Was the researcher male or female? | Page 4 – positionality and philosophical orientation |
| 5. Experience and training | What experience or training did the researcher have? | Page 4 – positionality and philosophical orientation |
| *Relationship with participants* | | |
| 6. Relationship established | Was a relationship established prior to study commencement? | Page 4 – positionality and philosophical orientation |
| 7. Participant knowledge of the interviewer | What did the participants know about the researcher? e.g., personal goals, reasons for doing the research | Page 4 – positionality and philosophical orientation |
| 8. Interviewer characteristics | What characteristics were reported about the interviewer/facilitator? e.g., Bias, assumptions, reasons, and interests in the research topic | Page 4 – positionality and philosophical orientation |
| **Domain 2: Study design** | | |
| *Theoretical framework* | | |
| 9. Methodological orientation and Theory | What methodological orientation was stated to underpin the study? e.g., grounded theory, discourse analysis, ethnography, phenomenology, content analysis | Page 4 – study design; positionality and philosophical orientation |
| *Participant selection* | | |
| 10. Sampling | How were participants selected? e.g., purposive, convenience, consecutive, snowball | Page 5 – recruitment and data collection |
| 11. Method of approach | How were participants approached? e.g., face-to-face, telephone, mail, email | Page 5 – recruitment and data collection |
| 12. Sample size | How many participants were in the study? | Page 7 - self-report measures and student perspectives; community partners’ experiences |
| 13. Non-participation | How many people refused to participate or dropped out? Reasons? | Page 7 - self-report measures and student perspectives; community partners’ experiences. *Due to the nature of this study and lack of follow-ups, there were no dropouts.* |
| *Setting* | | |
| 14. Setting of data collection | Where was the data collected? e.g., home, clinic, workplace | Page 5 – recruitment and data collection |
| 15. Presence of non-participants | Was anyone else present besides the participants and researchers? | Page 5 – recruitment and data collection |
| 16. Description of sample | What are the important characteristics of the sample? e.g., demographic data, date | Page 7 - self-report measures and student perspectives; community partners’ experiences. *To protect anonymity, we did not collect demographic data beyond student status and workplace.* |
| *Data collection* | | |
| 17. Interview guide | Were questions, prompts, guides provided by the authors? Was it pilot tested? | Supplementary file |
| 18. Repeat interviews | Were repeat inter views carried out? If yes, how many? | No, not applicable to this study |
| 19. Audio/visual recording | Did the research use audio or visual recording to collect the data? | Page 5 – recruitment and data collection |
| 20. Field notes | Were ﬁeld notes made during and/or after the inter view or focus group? | Page 5 – recruitment and data collection. *Field notes were made during the interviews and focus group discussion.* |
| 21. Duration | What was the duration of the inter views or focus group? | Page 5 – recruitment and data collection |
| 22. Data saturation | Was data saturation discussed? | Page 5 – recruitment and data collection |
| 23. Transcripts returned | Were transcripts returned to participants for comment and/or correction? | Page 6 – statistical analysis |
| **Domain 3: Analysis and ﬁndings** | | |
| *Data analysis* | | |
| 24. Number of data coders | How many data coders coded the data? | Page 6 – statistical analysis |
| 25. Description of the coding tree | Did authors provide a description of the coding tree? | Page 6 – statistical analysis. *We borrowed from reflexive thematic analysis which is a qualitative tool that does not employ coding trees.* |
| 26. Derivation of themes | Were themes identiﬁed in advance or derived from the data? | Page 6 – statistical analysis. *Salient points were generated from the data.* |
| 27. Software | What software, if applicable, was used to manage the data? | Page 6 – statistical analysis |
| 28. Participant checking | Did participants provide feedback on the ﬁndings? | Page 6 – statistical analysis. *Member checking was not utilized.* |
| *Reporting* | | |
| 29. Quotations presented | Were participant quotations presented to illustrate the themes/ﬁndings? Was each quotation identiﬁed? e.g., participant number | Page 10 – limitations. *To protect anonymity, we did not record transcripts and did not have illustrative quotes to support salient points. This has been acknowledged in the limitations.* |
| 30. Data and ﬁndings consistent | Was there consistency between the data presented and the ﬁndings? | Tables 3-4 |
| 31. Clarity of major themes | Were major themes clearly presented in the ﬁndings? | Tables 3-4 |
| 32. Clarity of minor themes | Is there a description of diverse cases or discussion of minor themes? | Tables 3-4 |
