# Supplementary material for: A mixed-methods assessment of community-engaged learning in a Master of Public Health program
Source: SAGE Open Med. 2023 May 29;11:20503121231176637. doi: 10.1177/20503121231176637 (PMC10233587; doi:10.1177/20503121231176637)
Supplement: sj-docx-2-smo-10.1177_20503121231176637 – Supplemental material for A mixed-methods assessment of community-engaged learning in a Master of Public Health program [file sj-docx-2-smo-10.1177_20503121231176637.docx]

**Online student questionnaire - Evaluation of community-engaged learning**

**SECTION I: Practicum Placement Information**

1. Prior to entering the MPH Program, what was your level of experience in public health? *(Options: not experienced, somewhat experienced, experienced, and very experienced)*
2. What was the name of the host agency in which you completed your practicum placement? *(Open-text response)*
3. During your practicum placement, what did your primary role involve?

Epidemiology and Statistics

Health Promotion

Public Health Communication

Public Health Policy

Public Health Administration

Program/Intervention Development/Evaluation

Public Health Education

1. Outside of your primary role, what other areas of public health were you involved with? Select all that apply.

Epidemiology and Statistics

Health Promotion

Public Health Communication

Public Health Policy

Public Health Administration

Program/Intervention Development/Evaluation

Public Health Education

1. Did you participate in any of the following activities? Select all that apply.

Research

Presentations

Conferences

Community Outreach

Writing reports

1. How would you rank your level of engagement and involvement within the host organization? *(Options: needs improvement, satisfactory, good, very good, and outstanding)*

**SECTION II: Core Competencies**

This section evaluates your abilities gained through the Public Health Practicum Course regarding the core competencies for public health, as outlined by the Public Health Agency of Canada. The core competencies are organized under seven categories: public health sciences; assessment and analysis; policy and program planning; implementation and evaluation; partnerships, collaboration and advocacy; diversity and inclusiveness; communication; leadership. All seven categories are included in this survey.

You will be required to select the option that best represents your level of competency both **BEFORE** and **AFTER** the completion of the Public Health Practicum Course. Please base your responses on the following scale: 1 = needs improvement, 2 = satisfactory, 3 = good, 4 = very good, and 5 = outstanding.

*(Please note: Questions 1 through 36 will appear in a chart format, with a scale appearing to the right of each competency. Each question will have a response row for BEFORE and AFTER completion of the practicum course. An option to not answer the question will be provided with each question in the chart)*

Rank your competence and ability to:

**Public health sciences**

1. Demonstrate knowledge about the health status of populations, inequities in health, the determinants of health and illness
2. Demonstrate knowledge about strategies for health promotion, disease prevention and health protection, as well as the factors that influence the delivery and use of health services
3. Demonstrate knowledge about the history, structure and interaction of public health and health care services at local, provincial/territorial, national, and international levels.
4. Apply the public health sciences (behavioural and social sciences, biostatistics, epidemiology, environmental public health, demography, workplace health, and the prevention of chronic diseases, infectious diseases, psychosocial problems and injuries) to practice.
5. Use evidence and research to inform health policies and programs

**Assessment and analysis**

1. Recognize that a health concern or issue exists.
2. Identify relevant and appropriate sources of information including community assets and resources.
3. Collect, store, retrieve and use accurate and appropriate information on public health issues.
4. Analyze information to determine appropriate implications, uses, gaps and limitations.
5. Determine the meaning of information considering the current ethical, political, scientific, socio-cultural and economic contexts.
6. Recommend specific actions based on the analysis of information.

**Policy and program planning**

1. Describe selected policy and program options to address a specific public health issue.
2. Describe the implications of each option, especially as they apply to the determinants of health and recommend or decide on a course of action.
3. Develop a plan to implement a course of action taking into account relevant evidence, legislation, emergency planning procedures, regulations and policies.
4. Implement a policy or program and/or take appropriate action to address a specific public health issue.
5. Implement effective practice guidelines.
6. Evaluate an action, policy or program.
7. Set and follow priorities, in order to maximize outcomes based on available resources.
8. Fulfill functional roles in response to a public health emergency.

**Partnerships, collaboration and advocacy**

1. Identify and collaborate with partners in addressing public health issues.
2. Use skills such as team building, negotiation, conflict management and group facilitation to build partnerships.
3. Mediate between differing interests in the pursuit of health and well-being, and facilitate the allocation of resources
4. Advocate for healthy public policies and services that promote and protect the health and well-being of individuals and communities.

**Diversity and inclusiveness**

1. Recognize how the determinants of health influence the health and well-being of specific populations.
2. Address population diversity when planning, implementing, adapting and evaluating public health programs and policies.
3. Apply culturally relevant and appropriate approaches with people from diverse cultural, socioeconomic and educational backgrounds, and persons of all ages, genders, health status, sexual orientations and abilities.

**Communication**

1. Communicate effectively with individuals, families, groups, communities, and colleagues.
2. Interpret information for professional, non-professional and community audiences.
3. Mobilize individuals and communities by using appropriate media, community resources and social marketing techniques.
4. Use current technology to communicate effectively.

**Leadership**

1. Describe the mission and priorities of the public health organization where you work, and apply them in practice.
2. Contribute to developing key values and a shared vision in planning and implementing public health programs and policies in the community.
3. Utilize public health ethics to manage yourself, others, information and resources.
4. Contribute to team and organizational learning in order to advance public health goals.
5. Contribute to maintaining organizational performance standards.
6. Build community capacity by sharing knowledge, tools, expertise and experience.

**SECTION III: Community-Engaged Learning**

This section evaluates your learning experience within a community-engaged setting, such as a **practicum placement**. Community-engaged learning synthesizes theoretical concepts with real world practice. The MPH program relies on host organizations to provide practical public health training that supplements in-class content in order to create graduates that are well-equipped to work within the public health field.

You will be required to indicate how the following skills have developed during your practicum placement. Please share your responses on the following scale: 1 = unsure, 2 = did not improve, 3 = slightly improved, 4 = improved, 5 = really improved.

**(Note**: **Questions 1 through 7 will appear in a chart format, with a scale appearing to the right.)**

1. Written Communication
2. Verbal Communication
3. Interpersonal Skills
4. Leadership Skills
5. Critical Thinking
6. Problem Solving
7. Self Efficacy
8. Did prerequisite courses adequately prepare you for your placement? *(Options: yes, no, unsure)*
9. Did this style of learning increase your understanding of in-class content? *(Options: yes, no, unsure)*
10. Did this style of learning improve retention of in-class content? *(Options: yes, no, unsure)*
11. Do you feel like this experience will be helpful for future opportunities in public health and the transition into the workforce? *(Options: yes, no, unsure)*
12. Do you think more classes should incorporate a community-engaged learning experience into the curriculum? *(Options: yes, no, unsure)*

Thank you for completing our survey! Your time and responses are greatly appreciated and will be used to ensure continuous improvement of the MPH program.

If you are willing to participate in a focus group to discuss and expand upon some aspects of community-engaged learning within the MPH program, please provide your name and email address.

**Focus group question guide – Evaluation of community-engaged learning**

1. What was the name of the agency you worked for, and what was your role there?

2. How would you describe the communication with your host agency? How satisfied were you with the relationship you developed with your host and the community?

3. Was working with a host organization a beneficial experience? How did you benefit:

a. Academically?

b. Professionally?

c. Personally?

4. Were there any drawbacks to working with a host agency?

5. Prerequisite courses are designed to prepare you for the practicum placement. In retrospect, were there any skills or gaps in knowledge you wish the prerequisite classes had better equipped you with prior to entering the placement?

6. The learning outcomes for this course are derived from the core competencies of public health. Which core competencies did you develop most during your practicum?

7. Are there competencies you wish you obtained more exposure to or skills you would have liked to further develop? Explain.

8. Do you think there are advantages to community-engaged learning that cannot be obtained from the in-class portion of your degree?

a. Explain how working in an experiential learning setting impacted the learning process. Was it accelerated? Enhanced? Distinct?

9. Finally, do you think your host agency learned anything from mentoring you? Explain.

**Semi-structured interview guide – Evaluation of community-engaged learning**

1. How many MPH students did you host over this last practicum period?

2. What role(s) do students typically hold while working at your organization? (e.g., epidemiology, health promotion, health communication, public health policy, program evaluation)

3. What are some of the benefits your organization receives from hosting MPH students?

a. Do you personally feel as if there is mutual knowledge transfer between the mentor and the student?

4. Are there any drawbacks to hosting MPH students?

a. Operationally, would you change any aspect of how this course is conducted?

5. What elements do you feel are essential to fostering a mutually beneficial partnership?

6. How/what do you think students learn differently in a community-engaged setting vs. in-class lecture?

7. Do you notice any changes in students from when they first enter to when they exit this experience?

8. What skills do you believe students acquire or develop most while under your supervision?

9. Do you think a practicum placement is critical to the teaching and delivery of public health curriculum? Why or why not?

10. Do you feel there was a change in university-organization relationship after you started hosting students?

**Results from the self-reported skill development**


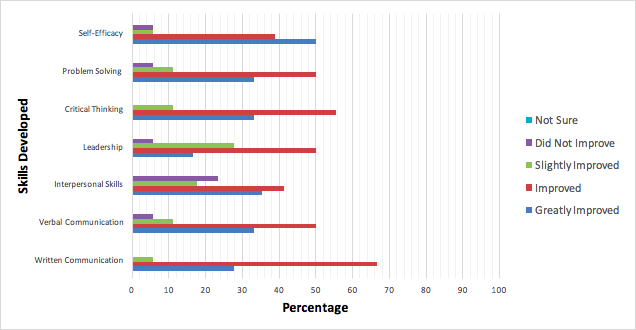


**Figure A1.** Master of Public Health students’ self-reported skills developed after completing a public health practicum.


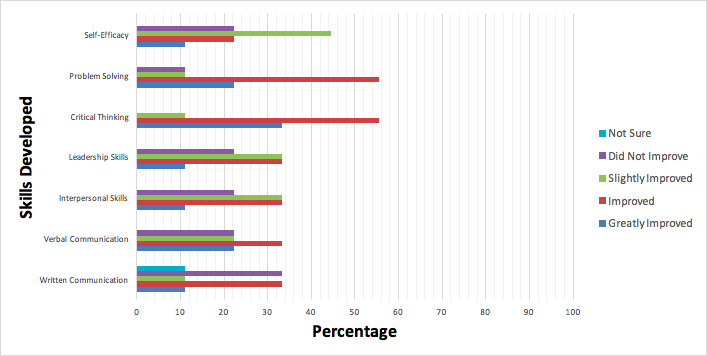


**Figure A2.** Master of Public Health students’ self-reported skills developed after completing in-class case studies.


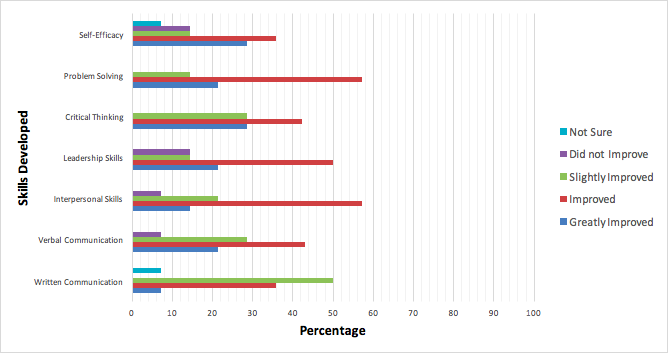


**Figure A3.** Master of Public Health students’ self-reported skills developed after completing a program development capstone assignment.


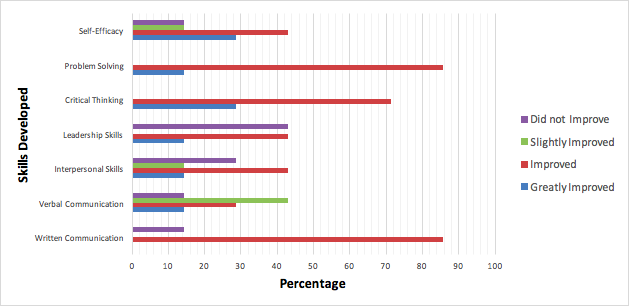


**Figure A4.** Master of Public Health students’ self-reported skills developed after completing applied public health research.
